# Should the Chairmanship Go Round?

**Published:** 1914-05-16

**Authors:** 


### THE EDITOR'S LETTER-BOX.

### Should the Chairmanship go Round?

*To the Editor of* The Hospital.

Sir,—No one can answer this question with a yea or nay. One thing only is certain: that it is a mistake to change the chairman every year, as is done by the L.C.C. By the time a man has got to know his work he is at the end of his term of office. If a man is chairman for only a year there is no honour attached to the position, and it is impossible for a man to make the job of which he is chairman his life's work. How many of your readers could tell who is the chairman of the L.C.C. to-day? If a chairmanship has to go round the whole committee, it must fall on men who are quite incompetent to be chairmen, however useful (or useless) they may have been as members of a committee.

On the other hand, many hospitals suffer from being under one chairman too long. I have in my mind a hospital to-day where the chairman is old, has been in power a long time, and who regards any proposal of improvement as a personal attack on himself. He is a "dear old man and we do not like to offend him," and so the hospital is drifting. Unless there is some rule or practice limiting the chairman's term of office, it is difficult, and rather invidious, to propose that some one else should be appointed. But, all the same, in no railway or big commercial undertaking that I know of has it been found advantageous that the chairmanship should "go round."

I suggest the following plan to all chairmen. There is a man to-day who holds one of the very highest positions in the country. He told me that he has made two men and one woman swear most solemnly that they will tell him if they ever hear people say that it is time he retired. It need not be their own opinion. He only asks them to repeat it if they hear it said of him. I write as one who has been chairman of a large hospital for eighteen years, and very much alive to the danger of being in that position too long for the good of the place, and I have adopted this plan myself so that I may have due warning when I am getting useless or in the way of a better man.—Yours faithfully,

Knutsford.

[The subject was raised in our News columns of May 2, page 117, with reference to the re-election of a chairman at Worcester General Infirmary.—Ed. The Hospital.]

### The Marriage of Faith and Science.

*To the Editor of* The Hospital.

